# Neglected Testing for Neglected Tropical Diseases at the CDC

**DOI:** 10.4269/ajtmh.22-0222

**Published:** 2022-04-12

**Authors:** Norman L. Beatty, Colin J. Forsyth, Robert H. Gilman, Davidson H. Hamer, Andrés F. Henao-Martínez, Natasha S. Hochberg, Jen Manne-Goehler, Rachel Marcus, Sheba Meymandi, Michael R. Reich, Adrienne Showler, Paula E. Stigler Granados

**Affiliations:** ^1^ Division of Infectious Diseases and Global Medicine, University of Florida College of Medicine, Gainesville, Florida;; ^2^ Drugs for Neglected Diseases Initiative, New York, New York;; ^3^ Johns Hopkins Bloomberg Boston Medical Center. School of Public Health, Baltimore, Maryland;; ^4^ Boston University School of Medicine. Department of Global Health, Boston University School of Public Health, Boston, Massachusetts;; ^5^ Boston Medical Center, Boston, Massachusetts;; ^6^ Boston University School of Medicine, Boston, Massachusetts;; ^7^ Division of Infectious Diseases, University of Colorado, Anschutz Medical Campus, Aurora, Colorado;; ^8^ Division of Infectious Diseases, Brigham and Women’s Hospital, Harvard Medical School, Boston, Massachusetts;; ^9^ Latin American Society of Chagas, Bristow, Virginia;; ^10^ Medstar Union Memorial and Good Samaritan Hospitals, Baltimore, Maryland;; ^11^ David Geffen School of Medicine at the University of California-Los Angeles, Los Angeles, California;; ^12^ Olive View-UCLA Medical Center, Los Angeles, California;; ^13^ Harvard T.H. Chan School of Public Health, Boston, Massachusetts;; ^14^ Division of Infectious Disease, Georgetown University Hospital, Washington, District of Columbia;; ^15^ Division of Global Health and Environmental Health, School of Public Health, San Diego State University, San Diego, California

In September 2021, the CDC suspended operations for diagnostic testing for most assays for parasitic diseases.^1^ The suspension covered more than 20 diagnostic tests for parasitic infections, including molecular and serological methodologies, morphological identification, and antimicrobial susceptibility testing (Table 1).^1^ As of April 7, 2022, only three of these services have resumed (serologic testing for Chagas disease, morphologic identification of parasites, and morphologic identification of malaria parasites). The CDC has not provided dates for resuming other tests.

**Table 1:** Diagnostic tests provided by the CDC Division of Parasitic Diseases and Malaria, unavailable as of February 17, 2022 (updated on April 7, 2022)

Test order no.	Test order name	Availability as of April 7, 2022
CDC-10234	Parasites: Morphologic Identification	***Available***
CDC-10237	Parasite—Special Study	Unavailable
CDC-10238	*Leishmania* Species Identification	Unavailable
CDC-10239	*Trichomonas* Antimicrobial Susceptibility	Unavailable
CDC-10456	Babesiosis Serology	Unavailable
CDC-10457	Baylisascariasis Serology	Unavailable
CDC-10458	Chagas Disease Serology	***Available***
CDC-10459	Cysticercosis Serology	Unavailable
CDC-10460	Echinococcosis Serology	Unavailable
CDC-10462	Filariasis Serology	Unavailable
CDC-10463	Leishmaniasis Serology	Unavailable
CDC-10464	Malaria Serology	Unavailable
CDC-10465	Paragonimiasis Serology	Unavailable
CDC-10466	Schistosomiasis Serology	Unavailable
CDC-10467	Strongyloidiasis Serology	Unavailable
CDC-10468	Toxocariasis Serology	Unavailable
CDC-10470	Trichinellosis Serology	Unavailable
CDC-10472	*Angiostrongylus cantonensis* Molecular Detection	Unavailable
CDC-10473	*Babesia* Molecular Detection	Unavailable
CDC-10475	Chagas Disease Molecular Detection	Unavailable
CDC-10477	*Cyclospora* Molecular Detection	Unavailable
CDC-10480	Malaria Molecular Identification	Unavailable
CDC-10481	Microsporidia Molecular Identification	Unavailable
CDC-10505	Fascioliasis Serology	Unavailable
CDC-10520	Malaria: Morphologic Identification	***Available***

Source: Centers for Disease Control and Prevention, 2022. *Currently Unavailable Test Orders*. Available at: www.cdc.gov/laboratory/specimen-submission/currently-unavailable.html. Accessed February 17 and April 7, 2022.

The CDC’s Infectious Diseases Laboratories provide diagnostic tests for many kinds of specimens, received from state public health agencies and other federal agencies. Private health-care providers (including individual clinicians) also submit specimens for testing through their local state health department laboratories for testing at no fee (to clinicians or patients).^2^ The full CDC Test Directory runs 644 pages.^3^ Many of these tests are not available at commercial laboratories. Some commonly ordered tests include assays for Chagas disease, strongyloidiasis, schistosomiasis, leishmaniasis, and cysticercosis. Suspension of these tests has had a direct negative impact on patient care.

There are many published examples where the CDC has played a critical diagnostic role for patients with parasitic infections in the United States. Some parasitic infections are non-endemic in this country, and clinicians often rely on the CDC to help diagnose cases primarily among immigrants to this country or travelers returning from abroad. Examples include paragonimiasis,^4^^,^^5^ gnathostomiasis,^6^ leishmaniasis,^7^ and fascioloiasis.^8^ The CDC laboratories also provide critical diagnostic services for parasitic diseases that are endemic but not common in the United States, including angiostrongyliasis,^9^ baylisascariasis,^10^ trichinellosis,^11^ and Chagas disease.^12^^,^^13^ Chagas disease molecular detection via polymerase chain reaction testing of blood and tissue samples is commonly used in patients who are immunocompromised, with concerns for reactivation of disease.^14^ This testing is time sensitive because of the life-threatening nature of the infection. The critical role of the CDC’s diagnostic capacity is illustrated by a recent report of acute Chagas disease reactivation in a patient after transplantation.^14^

On March 11, CDC officials notified some of us informally (by e-mail) that their goal is to resume polymerase chain reaction testing for Chagas disease and *Leishmania* species identification in March 2022, “though that may be delayed” (CDC, e-mail communication on CDC testing, March 11, 2022) and it has been delayed. This information has not yet been communicated to the public, and prospective dates for resuming other tests are still unknown outside the CDC. In addition, we do not know whether the CDC will resume all previously offered tests.

As strong supporters of the CDC and the Division of Parasitic Diseases and Malaria, we greatly appreciate laboratory support on neglected tropical diseases. However, as infectious disease clinicians and researchers, we are concerned about the interruption of laboratory services for parasitic diseases, now lasting more than 6 months. This interruption has far-reaching implications for patients, clinicians, and public health in the United States, as follows.^15^

First, the lapse in CDC laboratory services has created critical delays in diagnosis and treatment of patients with suspected parasitic infections.^15^^,^^16^ Many parasitic tests are not available elsewhere, or are only offered through certain commercial laboratories, many without specific parasitic disease expertise, sometimes at high cost, and sometimes with inferior quality relative to CDC tests.

Second, the lack of CDC laboratory services particularly affects vulnerable populations and underserved groups at the greatest risk for parasitic diseases, but with the least ability to pay. This has been particularly challenging for clinicians who care for and treat patients at high risk of Chagas disease,^15^ but also for clinicians caring for patients suspected to have other parasitic diseases, as noted earlier.

Third, the suspension of CDC laboratory services and lack of public alternative services have contributed to concerns about whether these services will be continued at the levels and scope needed to meet the needs of populations with suspected parasitic infections.

We acknowledge the regulatory complexity and costs of recertifying laboratory services, and the organizational and financial pressures created by the ongoing COVID-19 pandemic. We appreciate that the CDC is now making concerted efforts to communicate more effectively about its laboratory services. Nonetheless, we hope that the CDC’s Parasitic Diagnostic Laboratory will resume full operations for all previously available tests soon, and thereby again fulfill its critical function for parasitic disease diagnosis in the United States.

We hope this editorial galvanizes the CDC and concerned clinicians to work together to ensure our country’s long-term diagnostic systems for parasitic diseases. This high-priority problem of parasitic disease diagnosis deserves more attention, adequate financial resources at the CDC, and institutional support so that the vital services offered by the Division of Parasitic Diseases and Malaria can operate effectively and efficiently again.
